# Bladder cancer: A difficult problem?

**DOI:** 10.4103/0970-1591.38605

**Published:** 2008

**Authors:** Sudhir Rawal

**Affiliations:** Consultant Uro Onco-Surgeon, Rajiv Gandhi Cancer Hospital, New Delhi, India

According to the Delhi Cancer Registry, in 2003, bladder cancer was the 6^th^ most common cancer, surpassed in frequency only by cancers of the lung, larynx, tongue, prostate, and esophagus. Fifteen percent of all tobacco-related cancers are bladder cancers. Curiously, in Northern India this disease seems to affect younger patients. The majority of the patients present with superficial disease and are treated by transurethral resection of the bladder tumor. More than half of these patients experience recurrence, with about 20% progressing to muscle invasive disease. Outcomes in superficial and muscle invasive disease have improved over time. Intravesical chemotherapy has been found to prevent recurrence in superficial bladder cancer and the immediate postoperative instillation of mitomycin C has become the standard of care for small superficial tumors that have been resected completely without perforation. With improvements in surgical technique, and the evolution in urinary diversion procedure from ureterosigmoidostomy to orthotopic neobladder, the outcome in muscle invasive carcinoma of the bladder has improved remarkably with respect to both cancer control and quality of life.

This edition of the Indian Journal of Urology focuses on carcinoma of the bladder.

Theodorescu has discussed the molecular aspects of carcinoma bladder. The molecular level understanding of the mechanism of cancer genesis is increasing and carcinoma bladder is no exception. The authors have analyzed, at the molecular level, the factors responsible for two types of carcinoma bladder that have distinct behaviors. They discuss their potential implications (either therapeutic or prognostic) in the clinical management of the disease.

Dr. Jagdish N. Kulkarni has discussed in detail the changes in the staging of carcinoma bladder, with emphasis on the importance of cystoscopy and bimanual examination even in this era of MRI and CT scans.

Ashish Kamat (Prashant) has discussed the management of T1G3 bladder cancer and the role of radical cystectomy in these patients. They have also explored the role of molecular markers such as epidermal growth factor receptor (EGFR) in stage progression of non-muscle-invasive carcinoma of the urinary bladder and the potential therapeutic role of cell cycle inhibitors.

The article by Dr. Rakesh Kapoor of the Sanjay Gandhi Post-Graduate Institute of Medical Sciences (SGPGI), Lucknow, explores the role of BCG in non-muscle-invasive carcinoma bladder and its mechanism of action; he also discusses their experiences at the center.

Dr. Markand Kochikar has reviewed the existing literature in detail for his article on the role of surgery and adjuvant and neoadjuvant chemotherapy in the treatment of locally advanced and metastatic bladder cancer.

Marcus Kcukuz and Axel D. Merseburger have analyzed their results in 21 patients of T4 carcinoma bladder who were offered primary surgery in the form of radical cystoprostatectomy. Finally, there is the article by Dr. Deepak Jain from our center, which discusses the type of urinary diversion done after cystectomy in our country at various centers.

Invasive bladder cancer continues to be a very difficult disease to manage. All of us who are engaged in managing these individuals realize the limitations of aggressive therapy, as most of them do not live long and would succumb to bladder cancer. It is obvious that only surgical or radiation therapy is not the answer.

There is a need to develop better molecular markers, effective chemotherapy and hopefully a gene therapy to alleviate sufferings of patients who get cancer of bladder.

Finally, I take this opportunity to thank all my contributors who have helped me in organizing this symposium. I am grateful to the Editor, Indian Journal of Urology for having provided me this opportunity.

With best wishes for Happy New Year.

